# 

**Published:** 1852-06

**Authors:** 


### INDEX TO VOL. IX.

[THIRD SERIES.]

A

Acetate of lead, colica pictonum, 341
Acupuncture in fracture, - - 70
Amputation of lower jaw, - - 245
Anatomy, study of in Egypt, - 84
Animals, heat of - - - - - 87
Aorta compression of in Uterine
 Hemorrhage, - - - - 82
 Transformation embryonic, - 451
American Medical Association, - 409
 " " " 545
Apothecaries, duties of - - 429
Ague, common salt in - 452
Abdomen, wound of - - - 533
Acarus scabili, male - - - 156
Allen on Asclepias syriaca, - - 462
Anesthetics, their value, - - 507
Asclepias Syriaca, - - - 462
Artificial anus, - - - - - 533

B

Beasley's formulary, - - - 54
Beck's infant therapeutics, - - 57
Bell, Dr. John, resignation of - 274
Bibliograpical notices, - 456—459
Bladder bilobed, - - - - 73
Bowels, obstruction of - - 252
Bandage useless after delivery, - 521

C

Carpenter's physiology, - - 37
Cazenave on diseases of the skin, 319
Chloroform in phymosis, . - 267
 " on muscular fibre, - 346
 " in chorea, - - 450
 " in infantile convulsions, 451
Christian county vital statistics, - 369
Cimicifuga racemosa, - - - 77
Cod-liver oil in phthisis, - - 81
Colica pictonum, - - 266—341
Cox's companion, - - - 53
Cholera of 1848 in Russia, - 448
Chorea, by chloroform - - 450
Creasote in diarrhea. - - 289
Croup, tracheotomy, - - - 337
Calculi, containing a bean . - 525
Cerebral disease, - - - - 150
Chlorosis, - - - - - - 96
Chloroform, death from - 176, 500
Chloroform, external use of - - 536
Cod-liver oil, modus operandi - 183
Cold, polar, - - - - 165
 in Kentucky, - - 177
Constipation and chlorois - 96
Consumption, less mortal - - 272
Creasote, uses of - - - - 157

D

Dowler on muscular contraction, 61, 89
Drury's case of puerperal convulsions, - - - - - - 15
Darby on dysentery, &c., 409, 457
Drake on medical history, - - 220
 " on medical journals, - - 303
Doctors, advertising, - 443
Dickson on Life, &c., - - - 473
Dirt eating, - - - - 534
Dysentery, - . - 526, 531
Dropsy of the amnion, - - 529
Draper on respiration, - - 535

E

Edmond's vital statistics, - - 369

F

Fever, opium in - - - - 1
Fistula, easy cure of - - 258
 " impacted fæces, - 435
Folliculitis, - - - - - - 350
Fracture, ununited - - - 16
 " unconsolidated, - - 70
 " of cranium, - 203—249

G

Gregory's chemistry, - - - 29
Gadbury on the spleen, - - 464

Geology, to observe in, - - 117

H

Hardin on the cranium, - - 203
Hemorrhage, suppression of - 255
Hemorrhage, uterine - - - 82
Henry on large doses of opium, - 1
Hernia cerebri, - - - - 437
Hemoptysis and tubercle, - - 141
Hemorrhoids, - - - - 175
Hernia umbilical, - - - - 93
Honors to medical men, - - 449
Homœopathy, - - 331, 427

I

Inanition, - - - - - 86
Intermittent fever and common salt, 76
Iodine in atmosphere, - - - 347
Irvin's vital statistics, - - - 196

K

Kidney stone in - - - - 158

L

Larynx, polypus of - - - 248
Lawrence's cases of peritonitis, - 12
Lewis' thesis on disease, - - 292
Life insurance, - - - - 90
Lightning, death by - - - 89
Lying-in hospital Vienna, - - 149
Lemon juice in rheumatism, - 159

M

Malgaigne's surgery, - - - 34
Medical classes, - - 274, 361
Medicine, profession of - - 441
Meteorological table, - 92, 276; 368
Midwifery statistics, - - - 401
Mortality of states in U. S., - -
Muscular contraction, - - - 61
Medical attendance by the year, 182
evidence, - - - 540
reform, - - - - 495
witnesses. . . - 530
Menstruation vicarious, - - 153

N

New Orleans, mortality of - - 532

O

Ohio Medical Society reports, - 128
Oil of bitter almonds, poisoning - 260
Opium, large doses in fever - - 1
Opium eaters, - - - - 445
Opium in nervous diseases, - - 173

P

Pharmacy, report on - - - 429
Physicians and apothecaries, - 429
Paronychia, epidemic - - , 264
Parturition, bandage useless in - 521
Pereira's Materia Medica, - - 59
Peritonitis puerperal, - - - 12
Phthisis, cod liver oil in - - 81
" acute - ' - 277, 403
Placenta, ossification of - 261
Poisoning by bitter almonds, - 260
by santonine, - 268
Puerperal convulsions, - - 15
Pyroligenous acid a gargle, - - 434

Q

Quackery, new - - - - 164

R

Registration law of Kentucky, - 270
Respiration, new views on - - 535
Rheumatism, lemon juice in - 159
Rodgers Dr. J. K.'s case - - 101

S

Santonine a poison, - . - 268
Scurvy in Russia, - - 172
Shepherd on hernia, - - - 93
Shield's sore mouth of women. - 21
case of constipation, - 96
Skin, diseases of - - - 319
Sodium chloride in intermittents, 76
Silver, oxide of - - - - 471
Sore mouth of nursing women, - 21
Spleen, enlargement of - - 466
Spiritual Writings, - 273, 362, 453
Stomach ulceration, - - - 358
neutral sulphites, - - 439
Swain's midwifery statistics, - 401
Syphilis, non-mercurial treatment, 348
prophylaxis of - - 176
secondary - - - 469
Stone in kidney, - - - 158

T

Throat disease, - - - - 350
Tracheotomy, croup - - 337

U

University of Louisville, - - 361
Uterine hemorrhage, - - 82

V

Vagina, inversion of in labor - 83
Vegetables, heat of - - - 87
Vicarious menstruation,
Virulent matters in stomach, - 349
Vital statistics of Marshall county, 196
of Christian county, 369
of medical profession, 452
of Massachusetts, 130

W

Walker on marriage, - - - 55
Williams on ununited fracture, - 15
Withers on oxide of silver
Woodson on creasote
Writings, spiritual - - - 273

Y

Yandell, L. P.'s cases - - - 185
milk-sickness, - 374
Yandell, D. W. acute phthisis, 277, 403

### RECEIPTS OF MEDICAL JOURNAL FOR MAY, 1852.

| | |
|---|---|
| Dr. S. S. Watkins, Liberty, Texas, | $5 00 |
| Drs. Gadbury & Holmes, Benton, Miss., | 5 00 |
| Dr. Merrick. Freedom, Ind., | 5 00 |
| —— Barfield, Linden, Tenn., | 5 00 |
| —— Hoke, Beechland, Ky., | 5 00 |
| S. P. Hullehen, Wheeling, Va. | 17 95 |
| W. H. Lee, Columbia, Mo., | 5 00 |

---

**The WESTERN JOURNAL OF MEDICINE and SURGERY is published monthly by the undersigned, at the corner of Main and Fifth streets, Louisville, at $5 per annum, payable in advance. Each number contains 96 pages, making two volumes in the year of more than 550 pages each.**

**Contributions to its pages by the Physicians of the Valley of the Mississippi addressed to the Editors, are respectfully solicited.**

**Letters on business, to be addressed, postage paid, to the publishers.**

PRENTICE & HENDERSON.

